# Teaching central line placement: no clear window

**DOI:** 10.1186/cc13985

**Published:** 2014-07-10

**Authors:** Ryan Wilson, Sheeva Rajaei, Sugeet Jagpal, Amay Parikh

**Affiliations:** 1 Internal Medicine Division, Department of Medicine, Rutgers Robert Wood Johnson Medical School, 1 Robert Wood Johnson Place, PO Box 19, New Brunswick, NJ 08903-0019, USA; 2 Pulmonary and Critical Care Division, Department of Medicine, Rutgers Robert Wood Johnson Medical School, 1 Robert Wood Johnson Place, PO Box 19, New Brunswick, NJ 08903-0019, USA; 3 Divisions of Nephrology and Pulmonary/Critical Care, Department of Medicine, Rutgers Robert Wood Johnson Medical School, 1 Robert Wood Johnson Place, PO Box 19, New Brunswick, NJ 08903-0019, USA

## 

We appreciate Maizel and colleagues addressing the teaching of ultrasound-guided central line placement to medical residents [1]; however, we have several comments.

We feel that the success rates reported in this study are artificially high since most residents in 2014 are training with the use of ultrasound, and it is highly unlikely that the initial procedures being done by these senior residents (fourth and fifth year medical residents) were their first experience.

In addition, by teaching the landmark technique second, the residents at that time are definitely experienced with the procedure of central line placement. The shorter procedure time noted for the landmark technique is likely related to the fact the practitioners are more experienced at this point and they did not need to set up the ultrasound machine.

In addition, since procedure times were being measured, it would have been useful to separate out ‘difficult sticks’ [2]. It is anecdotally known that some patients have more difficult anatomy and require advanced maneuvers. Another possible variable that was not considered was the time of day of central line placement, since it has been established that central lines placed at night have higher complication rates than those placed during the day [3].

We feel that a better approach to answer the question of how to teach central line placement may be the concomitant approach: teach ultrasound-guided and landmark techniques at the same time to inexperienced practitioners, and then assess the success and complication rates.

## Authors’ response

Julien Maizel and Michel Slama

We thank Wilson and colleagues for their interest in our study and we would like to reply to their comments.

All procedures were performed during the day and the residents participating in the study were novices to central venous catheter (CVC) placement (fewer than three attempts). All residents were internal medicine residents and none were anesthesiology residents.

The success rate observed in our residents is in accordance with previous studies conducted with junior staff reporting success rates between 80 and 100% with the ultrasound-guided technique [4-6].

We agree with Wilson and colleagues that the shorter procedure time with landmarks demonstrates that residents acquired certain skills during the ultrasound-guided period. However, the success rate and the ability to puncture the vein were dramatically altered during the first landmark procedures and progressively improved after 10 landmark CVC placements. Learning the ultrasound technique alone therefore does not provide sufficient skills to perform an optimal landmark procedure.

The objective of this study was not to determine the best way to teach central line placement, but to determine whether our physicians who only learn the ultrasound-guided technique are able to perform the landmark technique with no specific training in this technique. It has been clearly established that the ultrasound technique is the technique of choice for both ‘easy stick’ and ‘difficult stick’ patients. Moreover, teaching the landmark technique is recommended only to ensure that physicians are able to perform CVC placement in emergency situations in the absence of an ultrasound machine and all of these emergency situations can be considered to be ‘difficult sticks’.

## Abbreviations

CVC: central venous catheter.

## Competing interests

The authors declare that they have no competing interests.
